# Energy Harvesting Chip and the Chip Based Power Supply Development for a Wireless Sensor Network

**DOI:** 10.3390/s8127690

**Published:** 2008-12-02

**Authors:** Dasheng Lee

**Affiliations:** Department of Energy and Refrigerating Air-conditioning Engineering, National Taipei University of Technology, Taipei, Taiwan, 106; E-Mail: f11167@ntut.edu.tw; Tel.: +886-2-2771-2171; Fax: +886-2-2731-4919

**Keywords:** Energy harvesting chip, Nano-ferrofluid magnetic core, Chip embedded transformer, Wireless sensor network (WSN), Packet loss rate (PLR)

## Abstract

In this study, an energy harvesting chip was developed to scavenge energy from artificial light to charge a wireless sensor node. The chip core is a miniature transformer with a nano-ferrofluid magnetic core. The chip embedded transformer can convert harvested energy from its solar cell to variable voltage output for driving multiple loads. This chip system yields a simple, small, and more importantly, a battery-less power supply solution. The sensor node is equipped with multiple sensors that can be enabled by the energy harvesting power supply to collect information about the human body comfort degree. Compared with lab instruments, the nodes with temperature, humidity and photosensors driven by harvested energy had variation coefficient measurement precision of less than 6% deviation under low environmental light of 240 lux. The thermal comfort was affected by the air speed. A flow sensor equipped on the sensor node was used to detect airflow speed. Due to its high power consumption, this sensor node provided 15% less accuracy than the instruments, but it still can meet the requirement of analysis for predicted mean votes (PMV) measurement. The energy harvesting wireless sensor network (WSN) was deployed in a 24-hour convenience store to detect thermal comfort degree from the air conditioning control. During one year operation, the sensor network powered by the energy harvesting chip retained normal functions to collect the PMV index of the store. According to the one month statistics of communication status, the packet loss rate (PLR) is 2.3%, which is as good as the presented results of those WSNs powered by battery. Referring to the electric power records, almost 54% energy can be saved by the feedback control of an energy harvesting sensor network. These results illustrate that, scavenging energy not only creates a reliable power source for electronic devices, such as wireless sensor nodes, but can also be an energy source by building an energy efficient program.

## 1. Introduction

The coming decade will see a widespread deployment of sensors throughout a variety of environments. In a built-in environment, benefits can be gained by deploying sensors. Sensor readings can be processed remotely or centrally, according to the architecture specification. Heating, ventilation, air conditioning, and lighting can be controlled by a centralized data center, and hence, a more optimized way of achieving extensive savings on energy consumption and personnel costs. It is obvious that building automation lowers the total cost of ownership, increases the security level, and raises the comfort of the people inside the building environment. Consequently, the building automation industry has seen remarkable growth in the last decades, and is still going through a rapid evolution [1, 2].

Recent technological advances in wireless communications have enabled easy installation of sensor networks. These wireless sensor networks (WSNs) are the base of a sensor-rich environment, referred to as “Ambient Intelligence” or “Smart Environments”. Although wireless communication offers many convenient features, the sensor node power supply, through either power lines or battery power, still presents obstacles in the development of WSNs. Wiring power to them seems ridiculous, since wireless communication is employed, and to date, most network nodes use alkaline batteries as energy sources. These batteries have fixed energy storage, which limits the sensor node's life time, and thus, they have to be replaced. For example, a Crossbow MICAz mode [3], operating at 1% duty cycle on standard 3000 milli-ampere-hour (mAhr) AA batteries, would require battery replacement every 17.35 months [4]. Often, the cost of physically deploying resources to change a node's worn out battery outweighs the cost of the node itself. To make matters worse, the waste battery causes serious environmental pollution.

Ambient energy harvesting is a possible breakthrough in the development of WSNs [5]. As electronic hardware becomes less expensive and smaller, more sensor applications are likely to appear, particularly as these miniaturized nodes offer the opportunity for the electronics to be embedded unobtrusively into everyday objects. A key issue for these wireless node designs is that they achieve high degrees of power-efficiency for autonomous and maintenance-free operation. Scavenging energy from renewable sources near the computing system is conceptually the best method for providing the node power.

Much work has been done in the field of the energy harvesting system development. The available harvesting technologies include solar cells, piezoelectric vibration generators, and energy from thermal sources. A new type of photovoltaic device, a dye-sensitized solar cell, has been studied to enhance light harvesting [6, 7]. Optical confinement [8] and light scattering effects [9] are found to be effective for these solar cells to improve light absorption efficiency, and therefore, increase energy harvesting efficiency. The possibility of converting the energy of environmental mechanical vibrations into useful electrical energy was explored [10]. A MEMS (Micro Electro Mechanical System) generator integrated with an ASIC power management circuit for powering wireless sensor nodes was reported [11]. The electromechanical transduction is performed using the piezoelectric effect of aluminum nitride thin films. The experimental results only prove the possibility of exploiting very low amplitude signals delivered by the MEMS generator. Harvesting of energy from heat sources can be achieved by the conversion of thermal gradients to electrical energy using the Seebeck effect. Reported results for power densities achieved from micro-fabricated devices are 4.5 μWcm^-2^ for a developed poly-SiGe device [12], and 10 μA of current, 30 μW of power generation for a patent pending low power thermoelectric generator device [13]. Above results relate to a temperature gradient/difference of 5K, which is typical for wearable applications and the environmental conditions. Higher temperature differences may be achievable in other environments, such as heaters in a building, but this is not applicable for the deployment of WSNs. Although it is possible to scavenge energy from several sources, solar energy is the most efficient energy source available for sensor networks [14]. Even for indoor applications, solar cells still have a power density of at least 0.5∼1 mWcm^-2^ under indoor 500 Wm^-2^ light intensity conditions, which is much higher than other energy scavenging competitors. From a review of energy harvesting technologies [15], typical data for vibration energy harvesting is 100 μWcm^-3^ under an acceleration of 1 ms^-2^; and for thermal is 60 μWcm^-2^ related to a 5K temperature difference.

In a review of energy harvesting technologies [15], energy scavenging for long-term deployable wireless sensor networks was discussed and the author announced that a truly autonomous, “deploy and forget” system could be achieved with an energy harvesting system. The “deploy and forget” scenario is attractive for the development of WSNs, however, the sensor module described in the case study of this review article still had rechargeable battery as a backup power source. Until now, most energy harvesting systems, either commercial products [16] or experimental prototypes [17-20], developed for mobile computing and WSNs play the role of auxiliary power sources for extending operational lifetime and rechargeable batteries are still necessary for the devices. In such a case, battery lifetime and charging from an unsteady energy source, such as indoor lighting, limits the number of allowable charge cycles, which are considerable issues facing the applications. WSNs consist of sensor nodes, powered by rechargeable battery, and cannot be considered as a “deploy and forget” system since the users must regularly maintain the battery. To make it worse, discharging the battery from energy scavenging has unstable characteristics, and thus decreases the battery lifetime.

Massive deployment of sensor nodes is a way to realize “Ubiquitous Computing” for a smart environment. However, massive deployment of sensor nodes with batteries would be an adverse situation due to the pollution from the spent batteries. Ubiquitous computing should not mean ubiquitous batteries. How to build a sustainable, renewable, and battery-less power solution for sensor nodes is a key challenge to be addressed in order to deliver future, remote, wireless, sensing systems and mobile computing devices.

In this study, an energy harvesting chip manufactured by a CMOS-MEMS processes [21] was developed to enable sensor nodes to use energy scavenged from solar cells. The power supply module consists simply of a solar cell, a capacitor, and an energy harvesting chip to provide a battery-less solution, and it is expected to be a sustainable and renewable power source for WSNs. The sensor nodes equipped with the chip based power supply were fabricated for performance tests, including sensor accuracy, response time, and signal deliveries of wireless communication. According to the test results, the feasibility of a “deploy and forget” system was investigated. The WSNs powered by energy scavenging were deployed in a convenience store as a case study. Based on a self defined protocol, the signal deliveries of wireless communication were tested in a real environment. The data loss rates of experiments and the case study were compared with the work of reference [22] to investigate the characteristics of the energy harvesting chip-based power supply. The WSNs were employed for air conditioning control in a convenience store, and the effects were discussed in detail in order to illustrate the potential application and the advantages of an energy harvesting power source.

## 2. Energy harvesting chip

**Figure 1.**
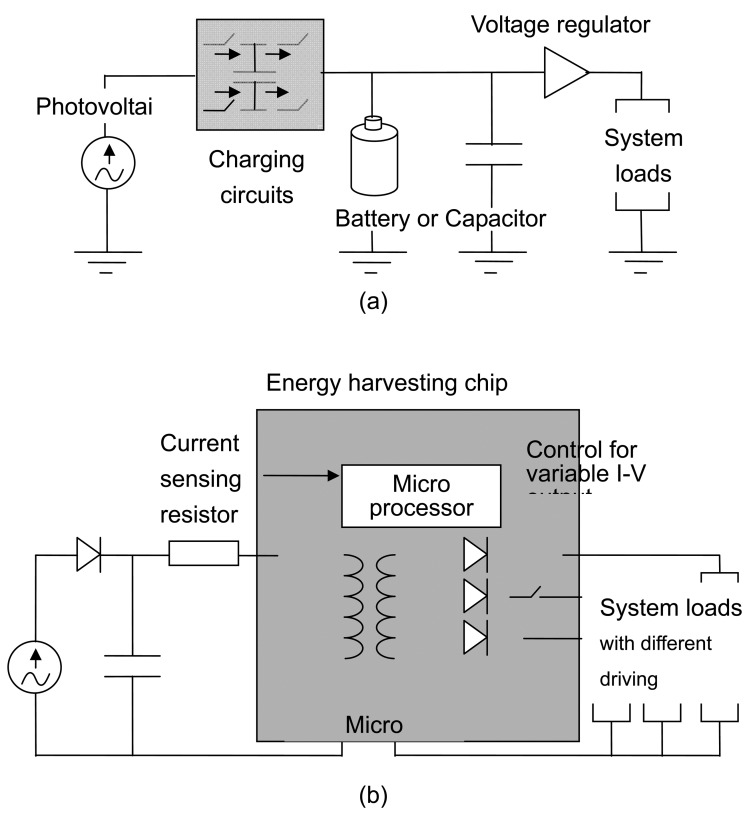
Electrical diagrams of **(a)** a traditional solar cell charging circuits, and **(b)** the novel power supply with the energy harvesting chip.

The energy-harvesting sources supply energy in irregular, random, and burst charging. The energy-harvesting system is required to capture and transfer intermittent low energy bursts. Switching regulators, which can boost the input voltage, are widely employed for stabilizing the output of the electronic power. The charging circuit depends on the nature of the input energy that drive the loads or store in the battery. Fully on or off switching devices alternately store and deliver the energy to the load via a combination of capacitor or battery. The traditional design of an energy harvesting system for photovoltaic can be referred to Figure 1a [14, 23].

The charging circuits must wait until sufficient energy is accumulated in the storage devices before attempting to transfer it to the load. The RC component consists of the load and the capacitor injects low frequency noise to the output, which affect the normal function of a wireless sensor node. Thus, the charging circuits are in turn normally connected with another regulator for stabilizing power output, as shown in Figure 1a. For driving multiple loads, such as WSN applications, the supplementary regulators or switching controllers not only increase the complexity of the charger but also decrease the efficiency of the energy harvesting system [23]. Moreover, since energy and power are invariably small, the system must ensure enough energy is harvested and stored before engaging in power-sensitive tasks. The battery is necessary for the traditional design of the energy harvesting system. As described above, such a system cannot be considered as a “deploy and forget” system since the users must regularly maintain the battery.

Comparing with the traditional charging circuits for solar cells, the energy harvesting chip can be illustrated by Figure 1b. Using the chip embedded magnetic component, a miniature transformer with micro-structured solenoids, it works as a multi-output constant voltage transformer. The isolated Buck-Boost topology [24] was employed for the energy harvesting chip due to its simple layout, less components required, and capacity for driving multiple outputs. By storing energy into the chip embedded transformer, the DC to DC conversions from photovoltaic to electric power can be stabilized. By adjusting the Duty Cycle of switching ON/Off, the amount of power transferred can be controlled. As the main switch connected to the primary side is turned on, all switches on the second side are cut off, and the transformer stores energy into the magnetic core of the micro-structured solenoids. Then the energy harvesting chip starts to partition its functions into time slices according to the time-division multiplex of the loads and solar cell powers by switching on the switches on the second side. The output voltage can be expressed by:

(1) Vo/Vi=nD/(1−D)

where n = N_2/_N_1_, the coil turns number ratio of the first and second sides. D is the duty cycle of the primary coil, Vi is the input voltage of the harvested energy, and Vo is the output voltage to the system load. The energy harvesting chip can sense the power input from the solar cell by the current sensing diode (CSR) and determine the duty for stabilizing the power output. With respect to the different loads, the voltage can be adjusted to meet variable power by changing the coil turns ratio and inducing currents from the secondary coil with optimized current gain. The component consists of the load, the capacitor, and the magnetic core, which filters the inherent switching waveforms of the circuits and the constant voltage outputs for the multiple load was expected. The stable driving voltages are helpful to the normal functions of wireless sensor nodes.

The energy harvesting chip is a system-on-chip [25] solution made for a simple power supply design. The energy harvesting power supply consists of a solar cell, a capacitor, and the chip, which provides a battery-less power system to the wireless sensor node to achieve the scenario of “deploy and forget”. Harvested energy stored by the capacitor and the chip embedded transformer can be used to meet the requirements of power-sensitive tasks. Moreover, the energy storage was expected to be used to extend the node lifetime during the period without using harvested energy. Different from the concepts of the traditional energy harvesting system, chip based energy harvesting did not focus on capturing more energy into the storage capacity, but dedicated to improving the power quality for the stable works of the sensor node and extending node lift time to keep usage data gathering for the sensor networks.

### 2.1. Chip embedded transformer made by CMOS-MEMS processes

**Figure 2.**
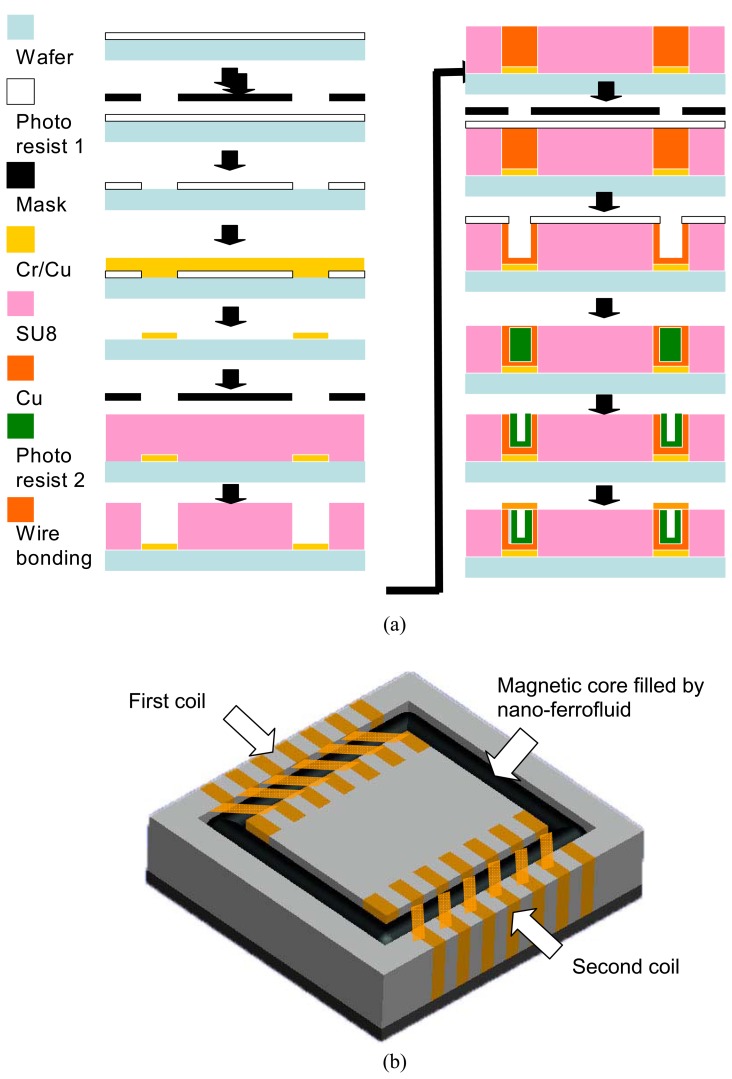
Post MEMS processes for manufacturing the energy harvesting chip **(a)** and the schematic view of the micro-structured transformer with the magnetic core filled by nano-ferrofluid.

The chip core is a miniature transformer consisting of a magnetic core and micro-structured solenoids made by a CMOS-MEMS processes. The integrated switching circuits and a mixed signal processor are fabricated based on the standard 0.5 m 2P2M CMOS processes. The post-CMOS micromachining processes include deposition of Cr/Cu metal layer, thick film process carried out with SU-8 photoresist, patterning the SU 8 thick film by the anisotropic dry etching, electroplating of Cu layer, forming the cavity by anisotropic wet etching and reaction ion etching (RIE) process for contact pad opening, and constructing the first and the second coils by copper wire bonding. In order to make the insulation, the wires were coated by liquid electric insulating paint. The manufacturing stages of the microstructure of the chip embedded transformer are summarized in Figure 2a.

### 2.2. Nano-ferrofluids magnetic core

Different from the magnetic film deposition processes reported in previous research [26, 27], nano-ferrofluids were employed for the formation of the high-permeability core of the chip embedded transformer. Capillary force drove the ferrofluids to enter the microchannel to form the flux closure of the primary and secondary coils. Figure 2b shows a schematic view of the miniature transformer with a nano-ferrofluids magnetic core. In order to obtain high permeability and low coercive force; a ferrofluid with a high nanoparticle volume concentration ratio was prepared by the chemical coprecipitation method. By mixing the solutions with ferrous ions and trivalent iron, magnetic nanoparticles are readily dispersed in a solvent. The chemical reaction can be expressed by:

(2) $${\text{Fe}}^{2+}+2{\text{Fe}}^{3+}+8{\text{OH}}^{-}\to {\text{Fe}}_{3}{\text{O}}_{4}+4{\text{H}}_{2}\text{O}$$

The oil-based nano-ferrofluids were prepared via reaction with NaOH, solvent cleaning with DI water, dispersion, and the solvent exchange process. Ferrofluids can be considered as tiny magnetized metal particles in an oil suspension and have near-zero hysteresis, which is the major cause of energy-waste in electric-power transformers. For uses in relatively small quantities, such as energy harvesting, high efficiency power conversion of the chip embedded transformer with nano-ferrofluids magnetic core can be expected. The design was developed and a patent was filed by our laboratory [28].

### 2.3. Variable voltage power supply for driving sensor node

Normally, the sensor node consists of several components, including the micro processor, radio frequency (RF) communication module, and different kinds of sensors. Each component has a specified driving voltage and current. With the chip embedded transformer, the energy harvesting chip can be used to drive multiple loads with variable power demands. In this study, a sensor node equipped with multiple sensors was developed to collect information about human body comfort degrees. The driving demands were analyzed and the chip based energy harvesting power supply was designed to meet the demands of variable voltage driving.

To obtain quantified information about thermal comfort in an indoor environment, the predicted mean vote (PMV) index was calculated [29]. It was necessary to know the temperature, relative humidity, and airflow. Therefore, an IC-based temperature sensor, a relative humidity sensor, a photo sensor, and a micro machined flow sensor were integrated on the sensor nodes.

The LM335 temperature sensor [30] was employed for temperature sensing over a temperature range of −55°C to +150°C. The IC-based sensor had a breakdown voltage directly proportional to its absolute temperature at +10 mV°K^-1^ When calibrated at 25°C the LM335 had typically less than 1°C error over a 100°C measurement range. This device consumed electric power of 300 μA at 3 V. The relative humidity sensing is performed by the Sensirion SHT1x [31], which displayed humidity values through a two-wire digital serial interface. Its relative humidity sensing accuracy was +/- 3.5%, within a range of 20% to 80%. The power consumption was 550 μA at 3V. The radiative temperature was estimated by the readings of the photo sensor [32]. Its accuracy level was +/- 2% of the reading range, which yielded 1.5 to 4.5 °C uncertainty. Power consumption of the infrared sensor and the signal processing circuits was less than 1 mA at 2.5V.

Indoor air flow greatly influences the degree of body comfort. Most cooling air conditioning energy saving schemes suggest using strong air convection to replace mechanical cooling. However, the body comfort flow is around 0.1 ms^-1^. The slow air speed detection was performed by a small sensor, requiring less power, previously developed in our laboratory [33]. The MEMS flow sensor measures low airflow speeds ranging between 0.1 ms^-1^ and 0.45 ms^-1^. The sensitivity of the flow sensor was indicated as 0.7 mVm^-1^s and 0.001 ms^-1^ velocity measurement resolution was achieved. The sensor was utilized to monitor the airflow conditions in a thermal environment. Analyzing the airflow speed required a 5 mA at 2.5 V power supply.

A microprocessor, PIC16F526, processed the sensor's input data and translated it into the packet-based messages for wireless transmission. The RF transmission module, coupled to a chip antenna, transmitted the data through a 935 MHz ISM band. The RF module and the microprocessor are driven by serial connection. The nominal power consumption is 6 mA at 3.5V and the transient power reached to 10 mA. Table 1 lists the power consumptions of each sensor and the signal processor with the RF transmission module.

A 50 mm × 55 mm photovoltaic cell module panel was mounted on a sensor node board to scavenge ambient light for the driving power. The solar cells were approximately 5-6% efficient, which was selected to allow the module to function at the light levels of 400-800 lux. With respect to the different driving voltages, the current supply from the photovoltaic cell was estimated by:

(3) $$\text{I}={\text{I}}_{\text{sc}}-(20.91\cdot {\text{I}}_{\text{sc}}+5.37\times 10^{-3})\cdot {\left(\text{V}/{\text{V}}_{\text{oc}}\right)}^{8}$$

where I is the current, V is the sensor nodes driving voltage; I_sc_ and V_oc_ are the short circuit currents and the open circuit voltage of the photovoltaic cell, respectively. The maximum short circuit current supply was 50 mA, and the open circuit voltage was up to 2.5 V.

The power supply based on the energy harvesting chip converts solar cell power to multiple voltage outputs to meet the driving demands of the components of the sensor node, as described above. Assuming 1500 lux ambient light and 80% conversion efficiency, the solar cell can provide enough power to the sensor node, and the design margin is approximately 100%.

### 2.4. Energy harvesting for effective data gathering

As described at the beginning of the section, an energy harvesting chip was designed to improve driving power quality and prolong sensor node lifetime for effective data gathering. Most research has focused on increasing the efficiency of the energy harvesting system and capturing more energy into the storage devices [1, 14, 15, 17-19]. However, the energy-harvesting sources supply energy is irregular, which thus yields unstable charging cycles. For rechargeable batteries, the battery lifetime, measured in charge cycles, varies from approximately 500 cycles for NiCd, Ni-MH, and Li-ion to approximately 300 for Li polymer [15]. The poor charging condition dramatically decreases the battery lifetime, and the waste batteries may cause serious pollution issues for the environment.

A capacitor could be utilized to store the energy, the lifetime of which, in terms of charge cycles, is orders of magnitude greater than batteries [34, 35]. However, the capacitor and even the ultra-capacitor discharge to empty within a short period without energy input, and can only be used as a buffer for energy storage but cannot provide the constant voltage output for the driving sensor node.

In this study, the energy harvesting chip, with the embedded transformer, was proposed to work with the capacitor to prolong the sensor node lifetime. The switching currents stored energy in the nano ferrofluid magnetic core, which was expected to maintain constant voltage output, as energy-harvesting sources interrupt energy supplies. The wireless sensor node can sense the harvested energy lowering the threshold by current sensing resistor (CSR), as shown in Figure 1(b). During this period, the current status and measured data can be stored in the non-volatile flash memory of the signal processor. The information would not be lost, even when without electric power. Waiting for the recovery of energy scavenging, the wireless sensor node can resume the works and deliver the previous data for ensuring effective data gathering.

### 2.5. Energy harvesting WSNs

Through the deployment of sensor nodes equipped with an energy harvesting chip, the energy harvesting WSNs was completed with a sustainable and renewable power source. The energy harvesting power supply is a battery-less solution to achieve the “deploy and forget” scenario. Although it's ideal for applications, how to synchronize the remote nodes becomes a problem since no backup power can be used to continuously generate the local clock signal.

A timing-sync protocol was proposed to achieve time synchronization in energy harvesting WSNs [36]. This protocol has two layers: synchronization and representation and the working principals are based on classified topology. Only one node is considered as layer zero, and is called the root node. Usually, a personal computer (PC) based data acquisition center is determined as root node. The neighboring nodes are classified as layer one, and the rest may be deduced by analogy. Once the structure has been established, the root node broadcasts a number of signals of clock time and the non-root nodes can be synchronized layer by layer. Afterward, the sensor nodes begin to send back measured data. The signal routing depends on the tree topology structured by time synchronization.

Since the study focused on the discussions of energy harvesting issues, the representation layer code was suggested as an easy format, with only 4 starting bits, 8 tempo time tag bits, 12 data bits, 2 bits for checksum, and 3 bits for ending the data stream. When the root awakens the node by representation packets, a wireless communication module on the remote sensor node will prepare to translate data. When the data is confirmed to be correct, this event is finished. If the received character does not include a stop code or the checksum is not correct, the data is required to be resent.

With an ARM code microcontroller chip and the 8-channel low voltage differential signaling device, an embedded system was constructed to perform the protocol-defined task of data communication services. Using a homemade communication module, the PC based data center was constructed to collect data from remote sensor nodes through RF communication. The data center collects data and calculates the transmission delay and response time by the following equation:

(4) $$\begin{array}{l} \text{Transmission delay}=\text{Time}\;{\text{tag}}_{\text{i}}-\text{Time}\;{\text{tag}}_{\text{j}}\\ \text{Response time}[i,j]=\frac{1}{m}\overset{m}{\underset{k=1}{\text{∑}}}\left(\text{Time}\;{\text{tag}}_{i,k}-\text{Time}\;{\text{tag}}_{j,k}\right) \end{array}$$

where *i* is the tempo time tag of the *i*-th node, j is the tempo time tag of root node at *j*-th requirement for data gathering of WSNs. and m is total data gathering number.

According to the feedback from the distributed sensor nodes, the PC based data acquisition center calculates PMV values by [29]:

(5) $$\text{PMV}=\left(0.303e^{-0.36M}+0.028\right)\left\{\begin{array}{l} \left(M-W\right)\\ -3.05\times 10^{-3}[5733-6.99\;(M-W)-P_{a}]\\ -0.42\left[(M-W)-58.15\right]\\ -1.7\times 10^{-5}M\;(5867-P_{a})\\ -0.0014M\;(34-t_{a})\\ -3.96\times 10^{-8}f_{cl}\;\left[{\left(t_{cl}+273\right)}^{4}-{\left(t_{r}+273\right)}^{4}\right]\\ -f_{cl}\;h_{c}\;(t_{cl}-t_{a}) \end{array}\right\}$$

where

tcl=35.7−0.028(M−W)−Icl{3.96×10−8fcl[(tcl+273)4−(tr+273)4]+fclhc(tcl−ta)}hc=〈2.38(tcl−ta)0.25for2.38(tcl−ta)0.25≥12.1Va12.1Vafor2.38(tcl−ta)0.25≤12.1Vafcl=〈1.00+1.29IclforIcl≥0.078m2CoW−11.05+0.645IclforIcl≤0.078m2CoW−1

M is the metabolic rate, and W is the external work. The parameter, f_cl_, is the ratio of a person's surface area while clothed, to the person's surface area while nude. That value was calculated by the thermal resistance of a person's clothing coefficient, I_cl_. These values could be estimated according the occupants' conditions in the thermal environment. The *ta* is the air temperature measured, *tr* is the mean radiant temperature, V_a_ is the air velocity, and P_a_ is the partial water vapor pressure. These measured parameters were provided by the distributed sensor node. The convective heat transfer coefficient, h_c_, is determined with respect to the air flow conditions in the space. By considering the measured parameters and the occupants' normal distributed conditions, the surface temperature of clothing t_cl_ is calculated, and the predicted comfort index PMV is determined.

The PMV indicators from many subjects are subjective environment conditions within certain environments, and finally are arranged with other factors, such as body movement, clothing, and environment to predict a possible comfort degree, which is divided into seven grades, from cold-3 to hot+3 and the neutral point is 0, suggesting a moderate condition. Table 2 shows PMV indicators and body feeling.

With the energy harvesting chip, the WSNs were expected to achieve real-time PMV monitoring by distributed sensor nodes. The chip base power supply provides a batteryless solution to the sensor nodes for realizing the “deploy and forget” scenario. Based on the feedback of energy harvesting sensor network, the air conditioning can be controlled to achieve optimized thermal comfort and save energy.

## 3. Experimental setup and case study

In order to verify the effectiveness of the energy harvesting chip and the concepts proposed in this study, the power conversion performances of the chip were tested and compared with traditional charging circuits. The sensor node powered by the energy harvesting power supply sent back data to a central data acquisition system with peer to peer mode and the response times of different sensing elements were measured, with respect to different ambient light intensity. The accuracy of each sensor was investigated. The WSNs were constructed and deployed in the convenience store for air conditioning control. The case study was arranged to test the practical operations of WSNs enabled by energy harvesting. How the battery-less system works, its effects for enhanced data communication, and control for optimized air conditioning control to save energy were discussed.

An 8 1/2 Digit Agilent 3458A Digital Multimeter was employed to test the voltage output characters of the energy harvesting chip. The chip was used to drive the sensor nodes with four different sensors, the signal processor, and the RF communication module. The voltage outputs from the energy harvesting chip were recorded by a digital multimeter under full-load conditions, meaning the sensor detected environmental temperature and the communication module continuously sent data back. The maximum power consumption was 5 mA at 2.5 V. The tests were performed under artificial light of 1,500 lux intensity, equal to the indoor light intensity of a convenience store, power input from the solar cell was approximately 15 mA at 2 V. Enough power supply from the solar cell for driving sensor node was assumed in the experiments. The solar cell charging circuits similar to the reference design presented in previous researches [14, 23] were also constructed for the comparison experiment. The voltage outputs from the charging circuits that enable the wireless sensor node, with the same experimental setup, were also recorded, and compared with the energy harvesting power supply. In order to investigate the characters of elongating a sensor node's lifetime, the power was suddenly cut off by disconnecting the power line of the solar cell and the transient output from the energy harvesting chip was observed. The results were also compared with the traditional switching circuits. Based on the comparisons, the advantages of the energy harvesting chip based power supply were discussed.

A Testo 400 multifunction instrument was employed to measure the thermal comfort parameters for a controlled experiment. The probes had been calibrated for adjustment capabilities of temperature measurement with an accuracy to <0.1 °C, relative humidity up to ± 1.0 %, and the velocity measurements in ± 1.0 % range of 0 to 20 ms-^1^. A DX-200 photometer was employed to test the accuracy of the photo sensor node. The instrument can detect light intensity with the range of 0 to 20,000 lux. The accuracy was ± 2.0 %. Comparing the measured data from the energy harvested sensor node and the commercial instruments, the accuracies of the data gathering by the energy harvesting WSNs were examined.

Since the sensor node functions according to the loads and the power input, the transmission delay caused the data to arrive late in different time periods, with respect to the amount of harvested energy, and thus yielded variable communication speed to the WSNs. The response time of different sensor nodes were measured under the conditions where the ambient light intensity was controlled from 250 to 2,400 lux. The experiments used peer to peer mode to evaluate the properties of communication. The packet loss rate (PLR) was proposed as the index for judging the communication quality [37]. It can be calculated by:

(6) PLR=Data receiving events/Request events

The transmission delay was continuously monitored to continue gathering data in synchronized time. The response time was calculated by averaging delayed time of each node. An adequate response time should be within the second order to meet the target of the real time PMV monitoring [38].

The WSNs was deployed in a convenience store as a case study. The energy harvesting chip and the chip based power supply can deliver remote environmental monitoring data, whereby wireless sensor nodes can be deployed in numbers for long periods of time. This requirement is only with solar cells, but not in line with the utilization of batteries. That is to facilitate the long term, large-scale deployment of millions of sensors, which do not need replenishing of the energy source during the lifetime of the monitoring program – that is “deploy and forget”. Four energy harvested sensor nodes were distributed in a 3.2 m × 5.95 m displays area to collect thermal comfort information of the customers in the store. The communication status of the WSNs was investigated by PLR index and compared with the results obtained from the previous researches of WSNs [22, 39]. This case study focuses on verifying the works of the energy harvesting chip for effective data gathering, and discovers a sustainable and renewable power source to enable WSNs for air conditioning control.

## 4. Results and Discussions

The energy harvesting chip was taped out in standard CMOS processes, and the microstructures were fabricated for the chip embedded transformer by post MEMS process. Integrating with the solar cell, the chip, and the related circuits, a power supply module can provide multiple voltage output to the wireless sensor node by scavenging energy from indoor light.

**Figure 3.**
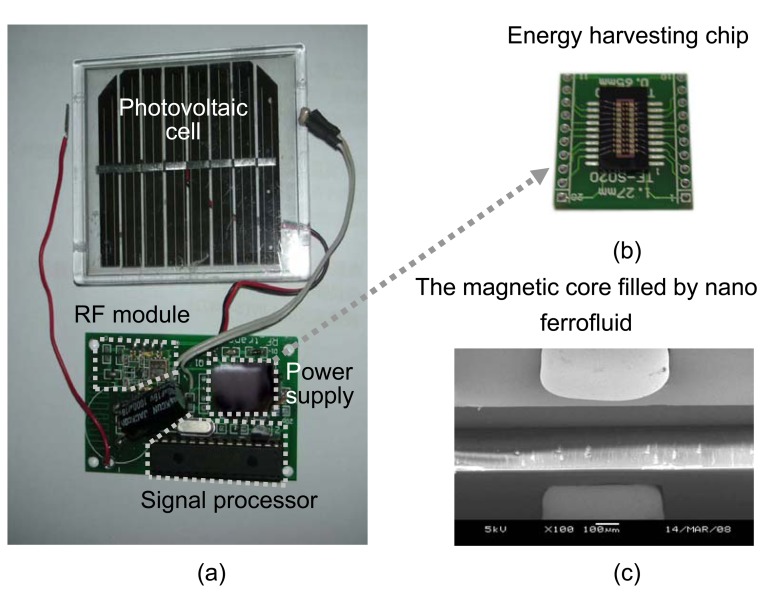
The pictures of sensor node with the energy harvesting power supply module (a), the energy harvesting chip core (b), and the SEM picture of the magnetic core (c) of the chip made by post MEMS processes.

Figure 3 shows the prototype of the energy harvesting power supply, the taped out chip, and the SEM picture of the trench on silicon for the formation of the magnetic core of the transformer. The system on chip provides a simple power solution and minimizes the size of the power supply. As shown in Figure 3a, the occupied area of the power module on the board of the sensor node is even smaller than a microprocessor for sensor signal processing. The energy harvesting chip was mounted on a ceramic platform and was then packaged in a hybrid package. The hybrid package is hermetically sealed, as shown in Figure 3b. By filling the nano ferrofluids, the magnetic core was formed in the etched trench as shown on Figure 3c. The harvested energy can be stored in a capacitor and the magnetic core of the chip then converted to driving power by switching control.

**Figure 4.**
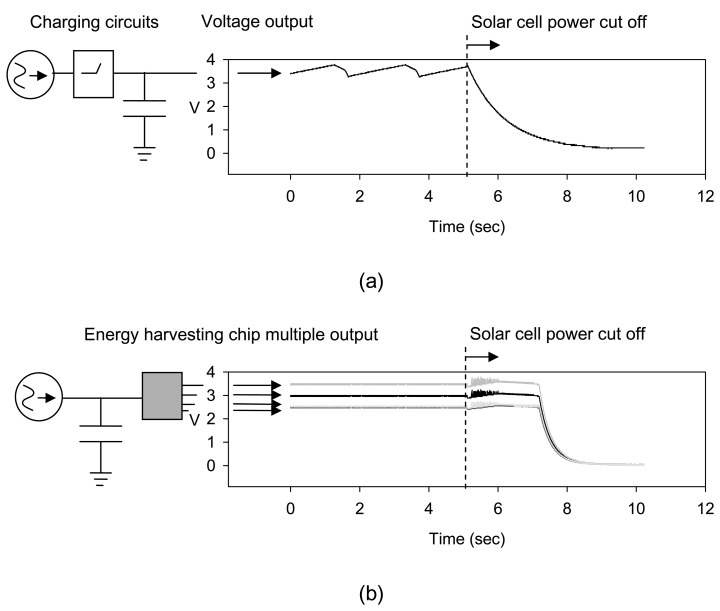
Voltage outputs from the traditional switching circuits used for solar cell charging (a), and the multiple outputs from the energy harvesting chip (b). The test condition is under the constant artificial light illuminating on the solar cell with the intensity of 1,500 lux.

The output characters of the energy harvested power supply were investigated and compared with the traditional switching circuits used for solar cell charging. Figure 4a shows the waveform of the voltage output from the charging circuits. As shown in the figure, the voltage output fluctuated with time under the condition of the power, from the solar cell, was under constant intense light, which is due to the switching control of the solar cell output. The capacitor and the driving load from the RC circuit inject low frequency noise to the driving voltage output. The fluctuated power supply to the sensor node may result in abnormal working of the electronic component. The voltage regulator is necessary to stabilize the driving the sensor node. Although it is assumed there would be enough power supply from the solar cell, the experimental results show that the solar cell efficiency is less than expected. No enough current density from the harvested energy could be used to drive the loads, and the battery has to be applied to the charging circuits design to store energy for the normal function of the sensor node.

Comparing with the charging circuits, the waveforms of the multiple voltage output from the energy harvesting chip, are as shown in Figure 4b. The constant voltage output from 2.5 to 3.5 V with oscillation of less than 0.5% was observed. The stable voltage output can be helpful for accurate detection by the sensor. The energy harvesting chip can partition its functions into time slices according to the time-division multiplex of the loads and feed each power through switching, and then the normal function of the micro processor and the communication module can be performed. The energy from the solar cell was stored by the capacitor and the magnetic core of the chip embedded transformer. The system worked similar to the RLC circuit, which consists of driving load, capacitor, and the inductive coil, and the inductive impedance was controlled for constant voltage output. The current density was enhanced by the nano ferrofluids magnetic core for driving the wireless sensor node.

It is noticed that the transient output of the energy harvesting chip could keep the driving voltage for a while after the solar cell power was cut off. The chip sensed the power drop of solar cell by CSR and changed both the switching control duty and the coil turns ratio to keep enough current density to enable the senor node. The driving voltages were controlled at the constant level within 5% offset. Sensor nodes can keep working for 2,300 ms under the experimental conditions. For the charging circuits, a dramatic voltage drop was observed due to the capacitor's rapid charging. This result indicates the magnetic core can be an effective buffer to the capacitor, and energy can be stored for elongating sensor node lifetime with no need of batteries. A battery-less power solution can be achieved.

With the elongated service lifetime, the sensor node can store the current measured results and the communication information in a non-volatile flash memory. The information would not be lost, even when without electric power. Waiting for the recovery of energy scavenging, the wireless sensor node can resume work and send back the previous data. The energy harvesting chip not only harvested energy, but also harvested data and stored in the memory for effective data gathering.

**Figure 5.**
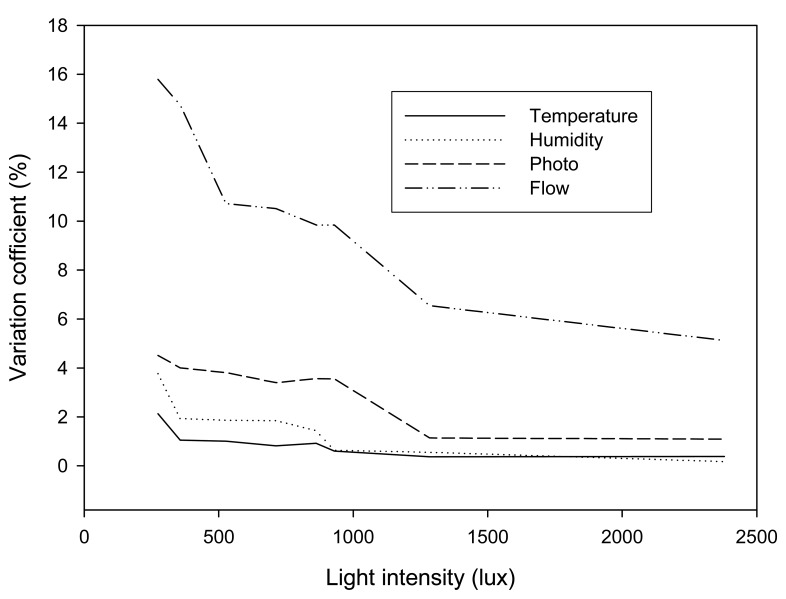
The variation coefficient of the measurement precision of the energy harvested sensor nodes with respect to different environmental light intensities.

Successful functions of a WSN require precise detection of sensor nodes. In a controlled environment of a laboratory, the sensing accuracies were investigated by comparing the measured results obtained by the energy harvested sensor node and commercial instruments. Figure 5 shows the sensor detection uncertainty, with respect to different environmental light intensities, ranging from 250 to 2,400 lux. The precision of the sensor nodes varied with the amounts of harvested energy. With respect to the environmental light intensity that exceeds 1,300 lux, the measurement uncertainties of the temperature, humidity, and photo sensors, as related to the commercial instruments, are less than 2%. The MEMS flow sensor, a homemade device, has a larger discrepancy than the other sensors. The uncertainties are within 6 to 8%, and it seems sufficient for the applications. Under the conditions of low environmental light, the measurement uncertainties of the energy harvested sensor node rose higher, due to the low amount of harvested energy. However, most sensors still can achieve accurate detection with the uncertainties of less than 6%, even when the environmental light intensity lowers to 240 lux, roughly the intensity of evening light. The flow sensor operated with low energy has up to 15% less accuracy than the instruments, but still can meet the requirements of the high-resolution airflow speed analysis for thermal comfort measurements. The PMV index calculated by Eq. (5) only concerns the flow speed variation up to 0.5 ms-1. Although there is a 15% uncertainty, the actual measurement discrepancy is only 0.0675 ms^-1^ and that has almost no influence in determining the value of PMV index.

**Figure 6.**
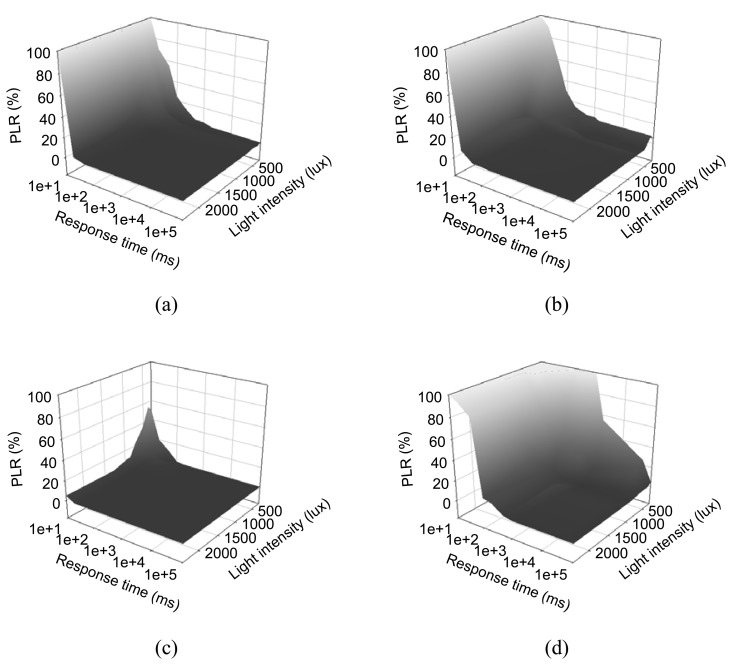
The packet loss rate, PLR, of temperature (a), humidity (b), photo (c) and flow sensor node (d) against the environmental light intensity

Communication speed and successful data delivery are both critical to WSNs. Under controlled environmental light, as described in the above section, the response times of the sensor node with four different sensors was calculated and the PLR was investigated. By decreasing the frequency of data request events from the data center, the response time of each sensor node becomes longer, which yields more successful data receiving events, and thus PLR can be increased. In Figure 6, the PLR of four sensors is plotted against environmental light intensity. As shown in Figures 6a-c, temperature, humidity, and the photo sensor node can provide almost 0% PLR with the response time of 100 ms under a light intensity of 1,500 lux. Regarding the flow sensor node, the PLR was higher than others', as shown in Figure 6d. A 2 % data loss was observed in.3% PLR was never reported in the previous studies, this indicates the PLR value still meets the the controlled environment of 1,500 lux light intensity. Although the high data loss rate, 2 to 12demands of the normal functions of WSNs. With 100 ms response time, all sensor nodes can deliver data with acceptable PLR. The data acquisition frequency of the energy harvesting WSNs was set to 400 Hz, and the data center could gather information by sequential communication with each sensor node.

**Figure 7.**
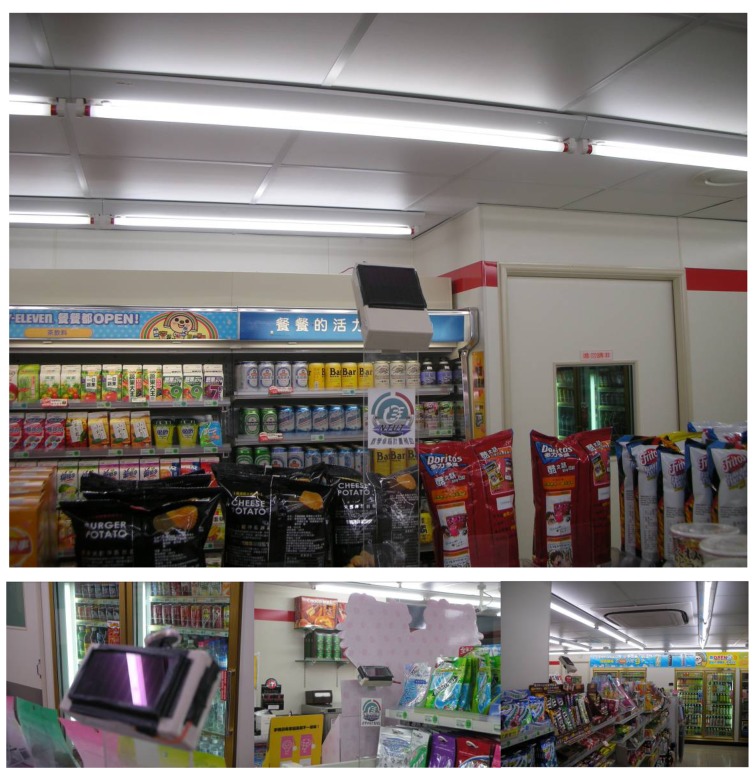
The energy harvesting sensor nodes deployed in a convenience store for thermal comfort measurement and optimized air conditioning control

A case study was performed in a practical environment. The energy harvesting WSNs were installed in a 24-hour convenience store. Figure 7 shows the sensor nodes deployed in the store. In order to enhance energy scavenging, the sensor node was assembled with illumination angles of 45 degrees. The averaged light intensity shining on the solar cell panel was 1,450 lux, which is close to the test conditions in the laboratory. Due to assembly location restrictions, only two sensor nodes could be equipped with a small size solar cell, with a panel area of 25 mm × 55 mm. Four sensor nodes distributed in the exhibition area collected the data of temperature, related humidity, luminous flux, and air flow speed of the test days. During one year of operation, the sensor network kept working to collect the thermal comfort information in the store. The energy saving control of air conditioning began to work with the energy harvesting WSNs after the year. During one month, the PMV data, as sent back by sensor nodes, was used to optimize the settings of two air conditioners. The PMV variations were recorded and compared with those just monitored by WSNs but not feedback controlled. The effects on energy saving were also investigated by electrical power consumption records.

**Figure 8.**
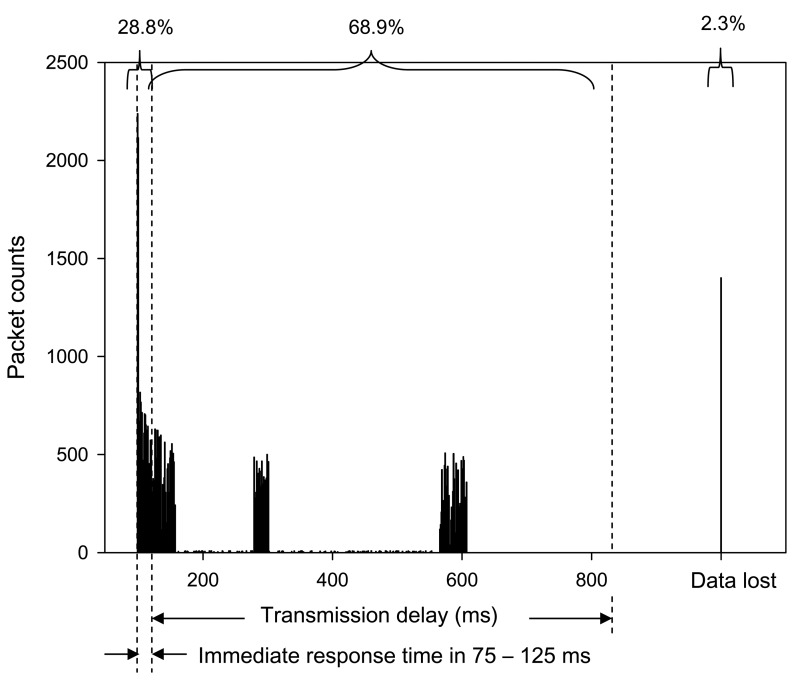
The number of packets delivered within the data acquisition response time, delayed counts, and lost counts recorded from a case study of the energy harvesting sensor network deployed in a convenience store.

During one month operation, the data communication status in the store was investigated by continuous monitoring of transmission delay, as expressed by Eq. 4. With respect to 400 Hz data requested commands, each sensor node delivers the packets to the data center within the response time of 100 ms. However, the data delivery may be interrupted by RF interferences or the sensor node loss of harvested energy. The data center can repeat requests to the sensor node to send back data if the packet is not complete, or can't be received within 25 ms time interval, which is the interval of the data acquisition frequency, thus data loss, due to RF interference, can be avoided as much as possible. In the store, the shielding caused by the occupants' motion may have created obstacles for the energy scavenged to the sensor node. With the energy harvesting chip, the senor node can have elongated service lifetime and the measured data and the communication status, such as received time lag, can be stored in non-volatile flash memory. The information would not be lost, even when without electric power. Waiting for the recovery of energy scavenging, the sensor node can resume work and send back stored data and current information to data center. Effective data gathering can be ensured, however, the packet may have variable delay times. Figure 8 shows the number of the packets delivered within the data acquisition response time, the lag counts, and the lost counts recorded in this case study. The packets that arrived late were counted and plotted against the lag. Data loss was denoted for the packets requested, but never received, by the data center. Analyzing the packet counts record, 28.8% packets arrived within a response time from 75 to 125 ms, 68.9% packets arrived late, and 2.3% packets were lost. Although most packets were within time lag, the measured data was still delivered, and the PLR is considered low at 2.3%. This value is compared to those reported in previous researches of WSNs, and the results illustrate that the sensor node enabled by the energy harvesting chip has good performance of data communication.

**Figure 9.**
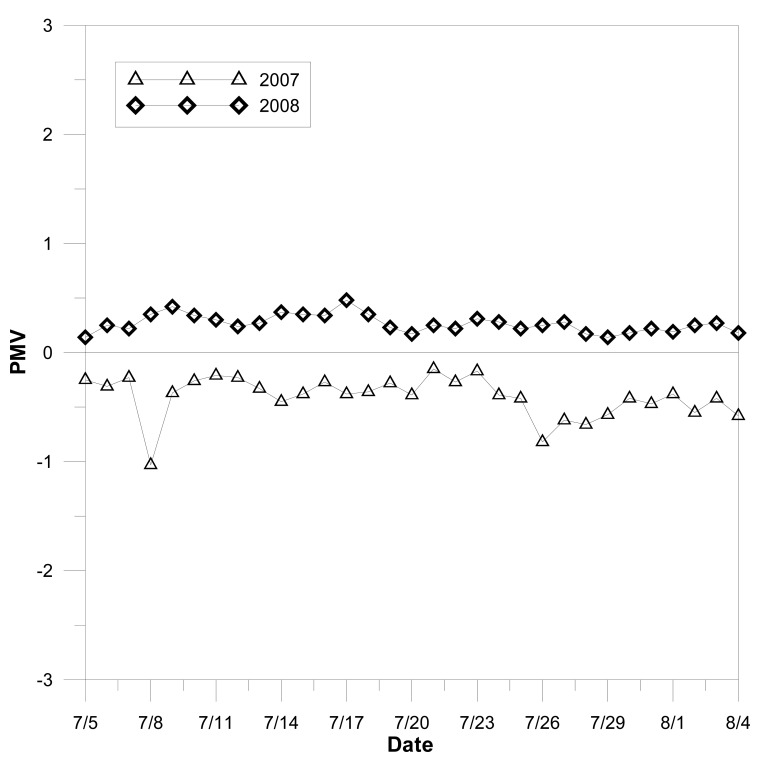
The PMV control in a convenience store based on the feedback of energy harvesting sensor network and its comparison to the PMV without control.

The temperature in the convenience store was adjusted and PMV was controlled by the feedback from the energy harvesting sensor network. All PMV data was obtained from real time monitoring of the sensor nodes, as enabled by the energy harvesting chip. One month PMV variations, as shown in Figure 9, were compared with the same month of the previous year. The PMV curve denotes the measurement in July 2008 as the control results; and the other curve denotes the measurement in July 2008, and is only monitored and without feedback controlled by the energy harvesting sensor network.

The smooth PMV curve obtained by the feedback control illustrates that thermal comfort in the store can be precisely determined, and that the sensor node is able to provide accurate detection of environmental parameters. This provides customers with a comfortable environment, and the energy consumption of air conditioning can be reduced.

**Figure 10.**
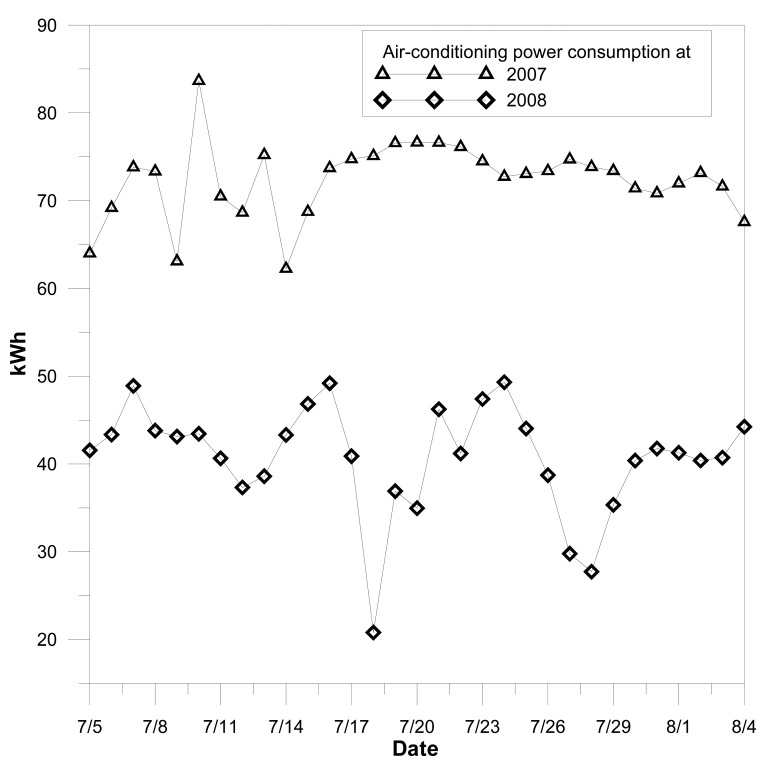
The electrical power consumption plot of air conditioning with and without PMV control based on the feedback of energy harvesting sensor network.

Figure 10 shows the electrical power consumptions of air conditioning with, and without, PMV control. Almost 54% energy can be saved with the feedback control of an energy harvesting sensor network. It has several potential applications, and the most important is that energy saving can be achieved by energy scavenging.

Although the chip enabled sensor node cannot work continuously without scavenging energy, the successful works of the energy harvesting sensor network were demonstrated in a 24-hour convenience store. Actually, most indoor applications of WSNs are in areas with occupancies and artificial light. WSNs normally work under sufficient light and can scavenge its energy for long term operation. The energy harvesting chip developed in this study enables a WSN to accurately detect and communicate efficiently. The advantages of the chip based power supply include variable voltage supply, multiple sensor driving, stabilized voltage output, and elongated service lifetime. It is a batteryless solution to wireless sensor nodes, and the “deploy and forget” scenario was demonstrated during one year practical operation. No more concerns regarding regular maintenance and battery replacement, and most important is no more concern over wasted battery pollution.

## 5. Conclusions

The energy harvesting chip was developed to scavenge energy from artificial light to supply power to a wireless sensor node. The chip, embedded with a micro structured transformer, was fabricated by CMOS-MEMS processes. Different from the magnetic film deposition processes, and nano-ferrofluids were employed for the formation of the high-permeability core of the miniature transformer. Capillary force drove the ferrofluids to enter the microchannel to form the flux closure of the primary and secondary coils. The harvested energy can be stored in the magnetic core and converted to variable voltage output for driving multiple loads. This system, on a chip, yields a simple, small, and more importantly, a battery-less power solution for a wireless sensor node.

Using a solar cell, the chip based power supply can provide constant voltage output for the stable works of the signal processor and the sensors equipped on the node. Under the status of no scavenged energy, the energy harvesting chip works to prolong the sensor node's lifetime. The driving voltages can be constantly controlled with little offset, and the working status and measured data can be stored in a non-volatile flash memory. The information would not be lost, even when without electric power, and while waiting for the recovery of energy input, the sensor node can resume work sooner. Instead of energy storage, the function of the energy harvesting chip aims to harvest data to retain normal operations of WSN.

The energy harvesting WSN was deployed in a 24-hour convenience store. During one year of operation, the sensor network powered by the energy harvesting chip kept working to collect environmental information and calculate the thermal comfort index PMV for the store. According to one month statistics, the communication quality index, PLR, can be as good as the results of WSNs powered by battery. The “deploy and forget” scenario was demonstrated. No more concerns over regular maintenance and battery replacement. A renewable and sustainable power source was exploited.

An energy harvesting WSN was employed to record customers' comfort degree in a store, and adjusted the environment inside the store. Based on the feedback of the distributed sensor nodes, air conditioning can be controlled to achieve optimized thermal comfort. In this way, the store can save almost half the electric power for air conditioning. Scavenging energy not only creates a reliable power source for electronic devices, such as wireless sensor nodes, but can also be an energy source through building an energy efficient program.

## Figures and Tables

**Table 1.** The power consumptions of each sensor and component of the wireless sensor node powered by energy harvesting.

**Item**	**Type**	**Power supply**	
Temperature sensor	LM355	300μA at 3 V	
Humidity sensor	SHT1x	550μA at 3V	
Photo sensor with signal processing circuits		1 mA at 2.5V	
MEMS flow sensor developed by this lab		5 mA at 2.5 V	
Signal processor and RF transmission module	PIC16F526	0.5∼10 mA at 3.5V	Nominal power: 20 mW Standby power: 1.75 mW Transient power max: 35 mW

**Table 2.** PMV indicators and body sensation.

**Index**	**Body sensation**

+3	Hot
+2	Warm
+1	Slightly warm
0	Neutral
-1	Slightly cool
-2	Cool
-3	Cold
